# Is *Toxoplasma gondii*‐secreted Protein With an Altered Thrombospondin Repeat (TgSPATR) a Potential Candidate for Immunisation? An Immunoinformatics‐Based Analysis

**DOI:** 10.1002/vms3.70807

**Published:** 2026-01-14

**Authors:** Masoud Foroutan, Elaheh Karimzadeh‐Soureshjani, Fatemeh Ghaffarifar

**Affiliations:** ^1^ Department of Basic Medical Sciences Faculty of Medicine Abadan University of Medical Sciences Abadan Iran; ^2^ Student Research Committee Abadan University of Medical Sciences Abadan Iran; ^3^ Department of Parasitology Faculty of Medical Sciences Tarbiat Modares University Tehran Iran

**Keywords:** bioinformatics, in silico, secreted protein with an altered thrombospondin repeat (SPATR), *Toxoplasma gondii*, vaccine

## Abstract

**Background:**

Toxoplasmosis is a widespread zoonotic disease that poses risks to pregnant women and immunocompromised individuals. Despite considerable efforts, no licensed vaccines are currently available for humans or animals. Rational vaccine design increasingly relies on immunoinformatics approaches to identify immunodominant epitopes and key immunological features.

**Objectives:**

This study aimed to characterise the *Toxoplasma gondii*‐secreted protein with an altered thrombospondin repeat (TgSPATR) using immunoinformatics tools to evaluate its suitability as a vaccine candidate.

**Methods:**

A comprehensive panel of bioinformatics servers was used to predict allergenicity, solubility, antigenicity, secondary and tertiary structures, post‐translational modification (PTM) regions, and B‐ and T‐cell epitopes, followed by in silico immune simulation.

**Results:**

TgSPATR consists of 534 amino acids with an estimated molecular weight of ∼57 kDa. The aliphatic index (65.71) and GRAVY score (–0.507) indicate moderate thermostability and an overall hydrophilic nature. Additionally, a total of 120 PTM sites were predicted, including phosphorylation, *O*‐ and *N*‐glycosylation, and palmitoylation sites. Secondary structure analysis (GOR IV, SOPMA, and NetSurfP‐3.0) revealed a predominance of random coils. Moreover, multiple servers (BcePred, SVMTriP, ABCpred, IEDB, ElliPro, and CTLpred) identified several high‐scoring B‐ and T‐cell epitopes capable of binding MHC class I and II molecules. According to SAVES v6.1, 74.1% and 93.8% of the residues of the initial and refined 3D models were located in favoured regions, indicating improved structural quality after refinement. In line with this, the ERRAT score also increased from 89.557 to 95.046. TgSPATR was predicted to be immunogenic and non‐allergenic. Finally, virtual immune simulation using the C‐ImmSim server showed that TgSPATR can elicit both humoral and cellular immune responses following three injections.

**Conclusions:**

This study provides foundational evidence that TgSPATR possesses key immunogenic properties and may serve as a promising vaccine candidate against acute and chronic toxoplasmosis. Nonetheless, wet‐lab experiments are required to validate these computational findings.

## Graphical abstract

*Toxoplasma gondii*‐secreted protein with an altered thrombospondin repeat was computationally characterised as an immunogenic, non‐allergenic protein carrying multiple B‐ and T‐cell epitopes. Structural validation and immune simulation suggest its strong potential as a vaccine candidate against toxoplasmosis.

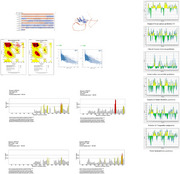

## 1 Introduction

It has been over a century since Nicolle and Manceaux discovered *Toxoplasma gondii* (*T. gondii*) within the tissues of the African rodent *Ctenodactylus gundi* (Dubey 2008). Toxoplasmosis is caused by *T. gondii*, an obligate intracellular protozoan of the phylum Apicomplexa. It ranks as one of the most common parasites worldwide and infects a wide range of warm‐blooded animals, including birds, livestock, and mammals (Dubey 2008). The parasite is found globally, and published reports indicate that over one‐third of the world's population is seropositive. It undergoes a sexual reproduction cycle in its definitive hosts, which are felines, whereas an asexual reproduction cycle occurs in intermediate hosts (Robert‐Gangneux and Darde 2012). There are at least three functionally distinct pathogenic forms, including tachyzoites, sporozoites (in oocysts), and bradyzoites (in tissue cysts) (Dubey et al. 1998). *T. gondii* can infect humans and other intermediate hosts through congenital infection, oocyst ingestion via the oral‒faecal route, and tissue‒cyst consumption (Robert‐Gangneux and Darde 2012). These findings also indicate that *T. gondii* infection can be transmitted through organ transplantation and, on rare occasions, through blood transfusion (Foroutan‐Rad et al. 2016; Khurana and Batra 2016).

Manifestations typically occur in hosts who are unable to mount an effective immune response. In humans, the predominant form of infection is typically latent and asymptomatic. However, under certain circumstances, such as in immunocompromised individuals or in congenitally infected foetuses and newborns, it can lead to significant health complications (Weiss and Dubey 2009). Ocular toxoplasmosis can occur owing to either congenital or acquired infections, arising from an initial acute infection or from the reactivation of a latent infection. In individuals with compromised immune systems, such as patients with AIDS, toxoplasmosis usually arises from the reactivation of a latent infection (Weiss and Dubey 2009). Therefore, toxoplasmosis is of great medical and veterinary importance (de Barros et al. 2022).

Chemotherapy is frequently used to treat toxoplasmosis. However, because the treatment exclusively targets tachyzoites and cannot eliminate bradyzoites within tissue cysts, its effectiveness is limited. Furthermore, this treatment also has side effects (Antczak et al. 2016). Therefore, the development of an effective vaccine against *T. gondii* is urgently needed. Significant progress has been made in the introduction of vaccine candidates that target key proteins of the parasite, especially dense granule antigens, rhoptry proteins, microneme antigens, and surface antigens (Foroutan et al. 2018b; Wang et al. 2019; Zhang et al. 2023). As Kawase et al. (2010) reported, *T. gondii*‐secreted protein with an altered thrombospondin repeat (TgSPATR) is a new member of the microneme protein family. This protein is secreted in a Ca^2+^‐dependent manner during the early stage of parasite invasion, is present on the outer surface of the parasites, and plays a crucial role in parasite invasion. In another study, Huynh et al. (2014) reported that TgSPATR plays a pivotal role in the invasion and virulence of the parasite. TgSPATR is expressed in tachyzoite, bradyzoite, and sporozoite forms (Huynh et al. 2014; Kawase et al. 2010). This protein is a homolog of *Plasmodium falciparum* SPATR (PfSPATR), and recombinant SPATR antibodies can suppress the invasion of sporozoites (Chattopadhyay et al. 2003). These observations support further investigation of TgSPATR as a promising vaccine candidate.

As a new interdisciplinary science, bioinformatics expertly uses mathematical, statistical, computational, physical, biological and medical tools to analyse biological data (Romano et al. 2011). These techniques are commonly used to evaluate gene and protein expression, predict protein structure, predict immunogenicity, and characterise general characteristics. Comprehensive analyses of the physical, chemical, and immunogenic attributes of proteins facilitate a deeper understanding of their structure and allow for the identification of promising vaccine epitopes (Flower et al. 2010; Members 2016). Employing bioinformatics approaches to identify protein epitopes is crucial for both diagnostic applications and vaccine development (Kazi et al. 2018). Since these methods offer several advantages, including time, effort, and cost efficiency, they are essential as a pre‐analysis step before undertaking wet laboratory evaluations (Flower et al. 2010; Foroutan et al. 2025b; Kazi et al. 2018; Romano et al. 2011). This research utilised a variety of online bioinformatics tools to predict the secondary and tertiary structures, physicochemical characteristics, post‐translational modification (PTM) sites, transmembrane domains, in silico immune simulation, and B‐ and T‐cell epitopes of the TgSPATR protein.

## 2 Methods

### 2.1 Sequence Accessibility

Initially, the complete TgSPATR amino acid sequence was acquired in FASTA format from the ToxoDB web server (Harb et al. 2020) under the ToxoDB number TGME49_293900. Table 1 lists the web addresses of all bioinformatics tools and databases used in this study.

**TABLE 1:** List of all bioinformatics online servers used in the research.

Server	Function	Setting	References
ToxoDB	TgSPATR protein sequence retrieval	N/A	Harb et al. (2020)
ExPASy ProtParam	Prediction of physicochemical parameters	N/A	Gasteiger et al. (2005)
NetPhos – 3.1	Prediction of phosphorylation sites in eukaryotic proteins	Default	Blom et al. (1999)
GPS‐Palm 1.0	Prediction of palmitoylation sites	Default	Ning et al. (2021); Ren et al. (2008)
NetNGlyc – 1.0	Prediction of *N*‐glycosylation sites	Default + Predict on all Asn residues	Gupta and Brunak (2002)
NetOGlyc – 4.0	Prediction of *O*‐glycosylation sites	Default	Steentoft et al. (2013)
AlgPred 1.0	Prediction of protein allergenicity	hybrid approach (SVMc + IgE epitope + ARPs BLAST + MAST)	Saha and Raghava (2006a)
VaxiJen v. 2.0	Protein antigenicity	Target organism: parasite (threshold: 0.5)	Doytchinova and Flower (2007)
ANTIGENpro	Protein antigenicity	Default	Cheng et al. (2005); Magnan et al. (2010)
SOLpro	Solubility upon overexpression	Default	Cheng et al. (2005); Magnan et al. (2009)
Protein‐sol	Protein solubility	Default	Hebditch et al. (2017)
DeepTMHMM	Prediction of transmembrane helices in proteins	Default	Hallgren et al. (2022)
Garnier–Osguthorpe–Robson (GOR) IV	Protein secondary structure prediction	Default	Garnier et al. (1996)
SOPMA	Protein secondary structure prediction	Default	Geourjon and Deleage (1995)
NetSurfP‐3.0	Prediction of the surface accessibility, secondary structure, disorder, and phi/psi dihedral angles of amino acids in an amino acid sequence	Default	Hoie et al. (2022)
SWISS‐MODEL	Protein structure homology modelling	Default	Guex et al. (2009)
GalaxyRefine	3D model refinement	Default	Heo et al. (2013); Ko et al. (2012)
SAVES v6.1	Structure validation server	Default	Colovos and Yeates (1993); Laskowski et al. (1993); Laskowski et al. (1996)
ProSA‐web	Prediction of the model's overall quality	Default	Wiederstein and Sippl (2007)
SVMTrip	Prediction of linear B‐cell epitopes	Default	Yao et al. (2012)
BcePred	Prediction of linear B‐cell epitopes, using physicochemical properties (hydrophilicity, flexibility/mobility, accessibility, polarity, exposed surface, and turns)	Default	Saha and Raghava (2004)
ABCpred	Prediction of B‐cell epitope(s) in an antigen sequence, using artificial neural network	Threshold: 0.75	Saha and Raghava (2006b)
		Length of the amino acids: 16	
		Overlapping filter: ON	
IEDB	Antibody epitope prediction from protein sequences	Default	Chou and Fasman (1978); Emini et al. (1985); Jespersen et al. (2017); Karplus and Schulz (1985); Kolaskar and Tongaonkar (1990); Parker et al. (1986)
ElliPro	Prediction of linear and discontinuous antibody epitopes based on a protein antigen's 3D structure	Default minimum score: 0.5	Ponomarenko et al. (2008)
		Maximum distance: 6 Angstroms	
MHC class I	MHC‐I binding predictions	Prediction method: ANN 4.0	Andreatta and Nielsen (2016); Lundegaard et al. (2008a); Nielsen et al. (2003)
		MHC source species: mouse	
		MHC allele(s): H2‐Db, H2‐Kk, H2‐Kb, H2‐Dd, H2‐Kd, and H2‐Ld	
		Length: 10	
MHC class II	MHC‐II binding predictions	Prediction method: SMM‐Align (NetMHC‐II 1.1)	Nielsen et al. (2007)
		Select species/locus: Mouse H‐2‐I	
		MHC allele(s): H2‐IEd, H2‐IAd, and H2‐IAb	
		Length: 15	
CTLpred	Prediction of CTL epitopes	Prediction approach: consensus	Bhasin and Raghava (2004)
		ANN cutoff: 0.51	
		SVM cutoff: 0.36	
		Tabular result: ten	
C‐ImmSim	In silico immune simulation	Simulation volume: 10	Rapin et al. (2010)
		Random seed: 12345	
		Simulation steps: 1050	
		Three injections at four‐week intervals with time series of 1, 84, and 168	

### 2.2 Evaluation of Physicochemical Characteristics

The ExPASy ProtParam tool was employed to determine various physicochemical attributes of TgSPATR (Gasteiger et al. 2005).

### 2.3 Antigenicity, Allergenicity, and Solubility Evaluation

To predict the antigenicity score, we used two servers, namely, ANTIGENpro (Magnan et al. 2010) and VaxiJen v2.0 (Doytchinova and Flower 2007). The AlgPred online server was subsequently used to predict the allergenicity of the target sequence. This server can predict allergenicity using six different approaches; we used the hybrid approach (SVMc + IgE epitope + ARPs BLAST + MAST), which has 85% accuracy at a threshold of –0.4 (Saha and Raghava 2006a). The protein solubility was also predicted using the Protein‐sol (threshold = 0.45) (Hebditch et al. 2017) and SOLpro (Cheng et al. 2005; Magnan et al. 2009) web servers.

### 2.4 Determination of Post‐Translational Modification (PTM) Regions of TgSPATR

The PTM sites of TgSPATR, comprising *N*‐glycosylation, *O*‐glycosylation, phosphorylation, and palmitoylation sites, were predicted using NetNGlyc – 1.0 (default setting + selection of all Asn residues) (Gupta and Brunak 2002), NetOGlyc – 4.0 (default setting) (Steentoft et al. 2013), NetPhos – 3.1 (default setting) (Blom et al. 1999), and GPS‐Palm 1.0 (settings: organism = mouse; threshold = low) (Ning et al. 2021; Ren et al. 2008) web servers, respectively.

### 2.5 Prediction of Transmembrane Domains

Using the DeepTMHMM server version 1.0.42 (09/10/2024) as a deep learning model for transmembrane topology prediction and classification, we predicted the presence of transmembrane domains in the TgSPATR protein sequence (Hallgren et al. 2022).

### 2.6 Prediction of Secondary and Tertiary Structures

To predict the protein secondary structure of TgSPATR, in terms of the probability of extended strands, *α*‐helices, and random coils (output width = 100 for both servers), we used the GOR IV (Garnier et al. 1996) and SOPMA (Geourjon and Deleage 1995) online platforms. The NetSurfP‐3.0 server was also used to predict structural disorder, solvent accessibility, and secondary structure, resulting in a detailed graphical representation (Hoie et al. 2022). The construction of three‐dimensional (3D) models is essential in reverse vaccinology. For this purpose, we utilised the SWISS‐MODEL online tool for homology modelling to predict possible 3D structures of TgSPATR (Guex et al. 2009). This approach is considered a standard method for building 3D structures of large proteins.

### 2.7 Refinement and Validation of the 3D‐Modelled Structure

The top model created by SWISS‐MODEL was selected and further refined via GalaxyRefine to improve the accuracy of the protein structure based on templates (Heo et al. 2013; Ko et al. 2012). The SAVES (structure validation server) v6.1 online tool was used to validate the three‐dimensional conformation of TgSPATR via PROCHECK (Laskowski et al. 1993; Laskowski et al. 1996) and ERRAT (Colovos and Yeates 1993). Additionally, the model's overall quality was checked via ProSA‐web (Wiederstein and Sippl 2007).

### 2.8 Prediction of Continuous and Discontinuous B‐Cell Epitopes of TgSPATR

The B‐cell epitopes of the TgSPATR protein were predicted via several servers. Initially, the SVMTrip server was used to predict continuous B‐cell epitopes (epitope lengths: 16, 18, and 20 aa) (Yao et al. 2012). Furthermore, BcePred was used to determine linear B‐cell epitopes by assessing physicochemical attributes with default settings. With a threshold setting of 2.38, the server achieves up to 58.7% accuracy, allowing prediction of epitopes based on flexibility, accessibility, exposed surface, hydrophilicity, polarity, and turns. We used the default values for prediction via the BcePred online server (Saha and Raghava 2004). Additionally, we employed the ABCpred server, which uses artificial neural networks (ANNs) to predict probable B‐cell epitopes within an antigen sequence. The server predicts epitopes with an accuracy of 65.93% using a recurrent neural network approach (Saha and Raghava 2006b). Prediction settings were adjusted as follows: B‐cell epitope length = 16 mer; threshold = 0.75; overlapping filter = ON.

The Immune Epitope Database (IEDB) server was used to predict several parameters, including hydrophilicity, Bepipred linear epitope prediction 2.0, antigenicity, surface accessibility, beta‐turns, and flexibility (Chou and Fasman 1978; Emini et al. 1985; Jespersen et al. 2017; Karplus and Schulz 1985; Kolaskar and Tongaonkar 1990; Parker et al. 1986). Finally, discontinuous B‐cell epitopes were predicted through the ElliPro online tool, which uses the protein's 3D model with default settings (maximum distance = 6 Å; minimum score = 0.5) (Ponomarenko et al. 2008).

### 2.9 Predicting T‐Cell Epitopes

Binding of SPATR to major histocompatibility complex (MHC) class I and class II proteins was assessed using the IEDB tool based on the alleles of the mouse strain. MHC‐I alleles included H2‐Db, H2‐Kk, H2‐Kb, H2‐Dd, H2‐Kd, and H2‐Ld. Predictions were made on 3/8/2025 using IEDB ANN (NetMHC ver. 4.0) for peptides 10 amino acids long (Andreatta and Nielsen 2016; Lundegaard et al. 2008a; Nielsen et al. 2003). MHC‐II binding predictions were performed on 3/8/2025 using IEDB SMM‐Align (NetMHC‐II 1.1) based on percentile rank sorting. MHC‐II alleles included H2‐IEd, H2‐IAd, and H2‐IAb, with peptide length = 15 mer (Nielsen et al. 2007). The CTLpred server was also used to determine probable cytotoxic T lymphocyte (CTL) epitopes using a consensus approach (Bhasin and Raghava 2004), with ANN cutoff = 0.51, support vector machine (SVM) cutoff = 0.36, and tabular result = 10.

### 2.10 In Silico Immune Simulation

The immunological profile of the protein (free of LPS) was assessed via the C‐ImmSim online server (Rapin et al. 2010). This server uses a position‐specific scoring matrix integrated with machine learning to forecast potential immune interactions. The results generated by this server reflect immunostimulatory activities across various anatomical areas, such as the lymph nodes, thymus, and bone marrow. Simulation settings were defined as follows: volume = 10, steps = 1050, random seed = 12,345, and three injections at 4‐week intervals (time points: 1, 84, and 168).

## 3 Results and Discussion

### 3.1 Physicochemical Attributes

This protein has 534 amino acids, with a molecular weight of 57,593.01 Da and a theoretical pI of 5.18, based on ProtParam outputs. This molecular weight, exceeding 5–10 kDa, suggests potential immunogenicity (Berzofsky 1993), and the theoretical isoelectric point indicates a weakly acidic pH at which the net charge is zero. Furthermore, the protein sequence contains 80 negatively charged residues (Asp + Glu) and 62 positively charged residues (Arg + Lys). The determined isoelectric points and charge characteristics offer important insights for selecting suitable buffer systems and refining expression conditions in subsequent experimental studies (Xia 2007).

The half‐life was estimated to be 30 h in vitro (in mammalian reticulocytes), over 20 h in vivo (yeast), and over 10 h in vivo (*E. coli*). The total number of atoms was 7946 (chemical formula: C_2472_H_3911_N_719_O_820_S_24_). The instability index of TgSPATR was calculated to be 63.63, indicating instability. It exhibits moderate thermotolerance, with an aliphatic index of 65.71, and displays hydrophilic characteristics, as indicated by a grand average hydrophilicity (GRAVY) score of −0.507. A negative GRAVY value indicates that the protein is hydrophilic, promoting stronger interactions with surrounding water molecules (Biro 2006). One key method for protein stability prediction is the dipeptide composition‐based instability index, which states that proteins with values below 40 are considered stable (Gamage et al. 2019). These advantageous physicochemical characteristics are critical considerations for the choice of expression vectors and purification experiments (Dey et al. 2014).

### 3.2 Antigenicity, Allergenicity, and Solubility Evaluation

AlgPred, based on a hybrid model, demonstrated that the TgSPATR protein is non‐allergenic. Antigenicity scores obtained from VaxiJen v2.0 (0.7601) and ANTIGENpro (0.7645) were both above the respective thresholds, supporting that TgSPATR is a probable antigen. According to SOLpro and Protein‐Sol, this protein was predicted to be soluble, with probabilities of 0.738 and 0.605, respectively (Figure 1A,B). These characteristics are important for vaccine development and diagnostic kit creation (Tork et al. 2025; Wang et al. 2016).

FIGURE 1(A) Solubility and (B) deviation from the population average, charge score, and fold propensity of *Toxoplasma gondii*‐secreted protein with an altered thrombospondin repeat (TgSPATR) predicted using the Protein‐Sol online server. Bioinformatics analysis of TgSPATR phosphorylation regions: (C) number of predicted sites for S (serine), T (threonine), and Y (tyrosine); (D) phosphorylation site prediction diagram; (E) transmembrane domain prediction, and secondary structure predictions using (F) GOR IV, (G) SOPMA, and (H) NetSurfP‐3.0 servers.

**Figure:**
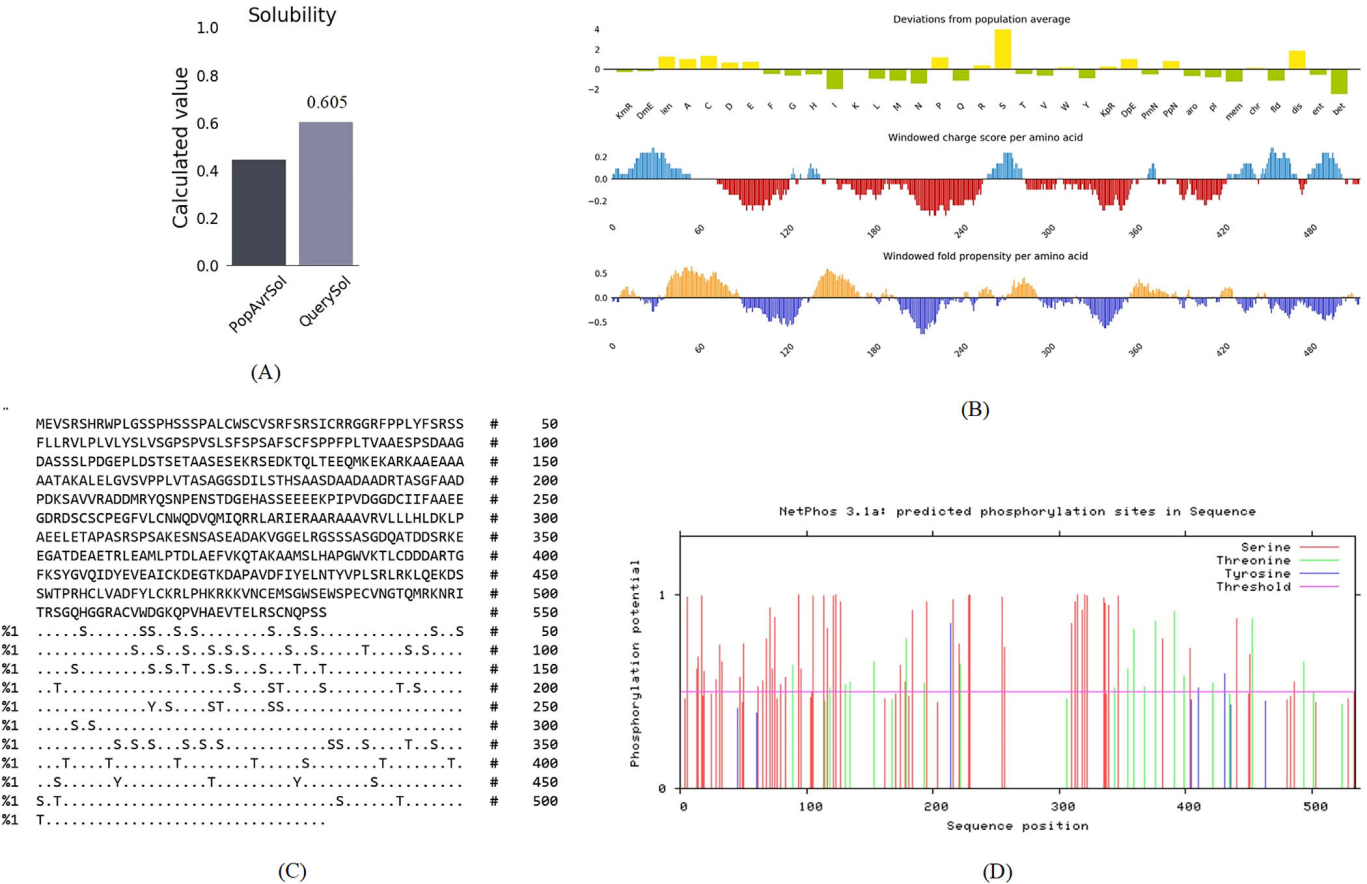

**Figure:**
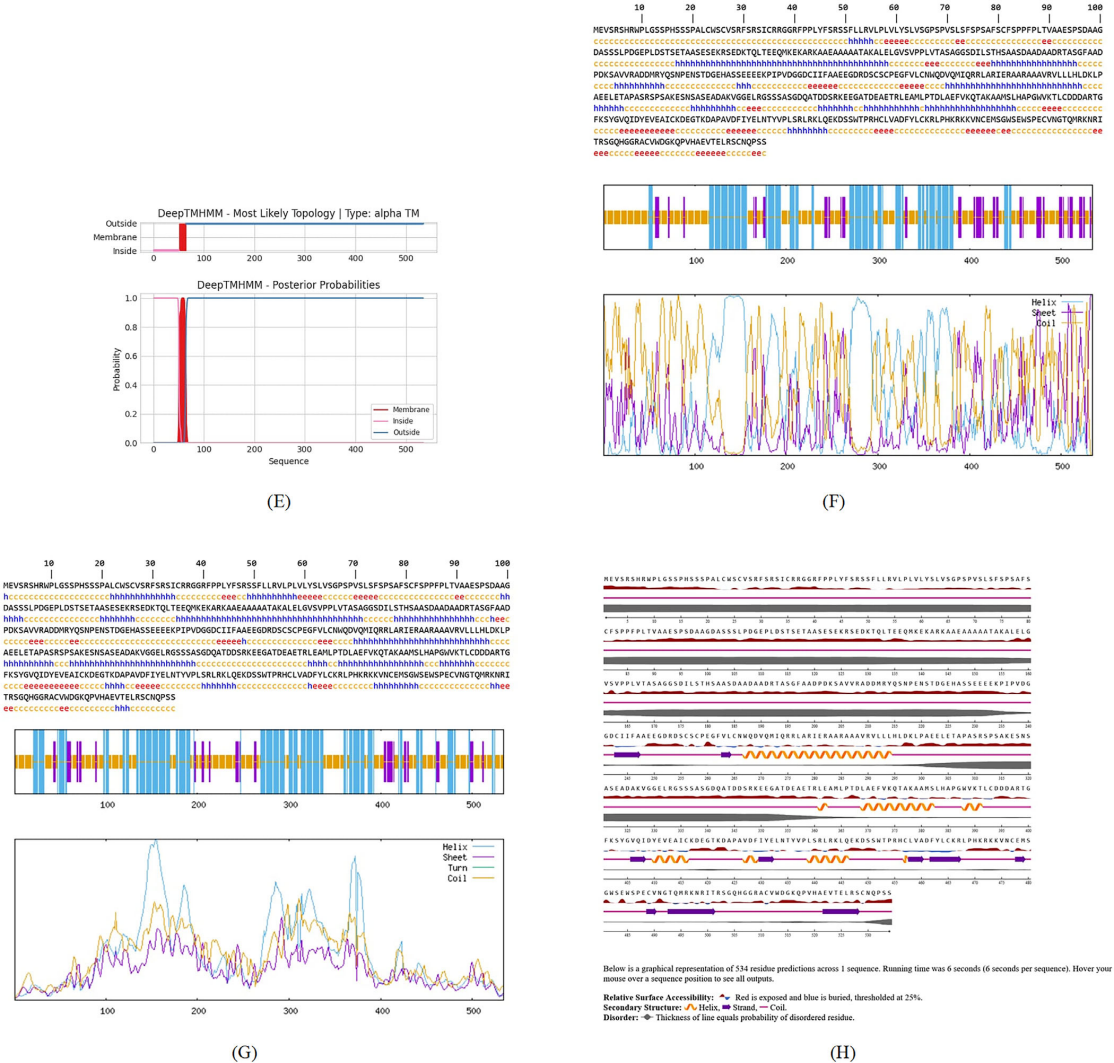

### 3.3 PTM Sites and Transmembrane Domain

PTMs are present in nearly all proteins and affect their function, playing vital roles in cellular regulation (Blom et al. 2004; Lee et al. 2009). A total of 120 PTM sites were identified, including 73 phosphorylation sites: 51 serine, 3 tyrosine, and 19 threonine residues (Figure 1C,D). Additionally, 38 *O*‐glycosylation sites were predicted at positions 4, 6, 14, 94, 96, 104, 105, 114, 115, 116, 118, 121, 123, 127, 131, 178, 184, 195, 216, 221, 222, 228, 229, 306, 310, 312, 314, 318, 320, 322, 335, 336, 337, 339, 347, 354, 501, and 503. Six *N*‐glycosylation and three palmitoylation sites were also identified at positions 217, 266, 319, 434, 476, and 491 (NetNGlyc) and via GPS‐Palm. These PTM regions may be crucial for protein activity and may impact recombinant protein production, favouring eukaryotic expression systems over bacterial ones (Lee et al. 2009; Walsh 2006). A transmembrane helix was identified from positions 51 to 64 (DeepTMHMM) (Figure 1E).

### 3.4 Structural Predictions

Identifying the secondary structure of a protein is a key determinant for assessing its overall three‐dimensional structure (Foroutan et al. 2018a). Secondary structure analysis using GOR IV, SOPMA, and NetSurfP‐3.0 revealed that random coils were the most common secondary structures, with 291 residues (54.49%) and 270 residues (50.56%) in GOR IV and SOPMA, respectively, followed by alpha helices, with 163 residues (30.52%) and 208 residues (38.95%) (Figure 1F,G). NetSurfP‐3.0 results confirmed these findings (Figure 1H). The presence of alpha‐helices and beta‐turns with strong hydrogen bond energy supports protein stability and may enhance antibody interaction (Foroutan et al. 2021). SWISS‐MODEL was used to construct 3D structures of TgSPATR. The best model had 99.81% sequence identity and coverage of 1.00 from residues 1 to 534 (Figure 2A,B).

FIGURE 2SWISS‐MODEL server output. (A) Model‐template alignment; (B) three‐dimensional (3D) structure of the *Toxoplasma gondii*‐secreted protein with an altered thrombospondin repeat (TgSPATR). Validation of the TgSPATR tertiary structure: analysis of the Ramachandran plot via PROCHECK for the (C) crude and (D) refined models revealed 74.1% and 93.8% of the residues in favoured regions, respectively. *Z* scores were calculated as −7.27 and −7.45 (E) prior and (F) post‐refinement, respectively, indicating improved 3D model quality after refinement. The ERRAT online tool was used to assess 3D model reliability. *On the error axis, two lines are drawn to indicate the confidence with which it is possible to reject regions that exceed that error value. **Expressed as the percentage of the protein for which the calculated error value falls below the 95% rejection limit. High‐resolution structures generally produce values around 95% or higher. For lower resolutions (2.5 to 3A), the average overall quality factor is around 91%. ERRAT scores for the crude (G) and refined (H) models were 89.557 and 95.046, respectively. (I) Propensity scale plots of TgSPATR, from top to bottom, show BepiPred linear epitope prediction, beta‐turn, surface accessibility, flexibility, antigenicity, and hydrophilicity. The favourable regions linked to the features of interest are indicated by yellow colours (above the threshold). The regions associated with the attributes of interest that are unfavourable are shown in green (below the threshold).

**Figure:**
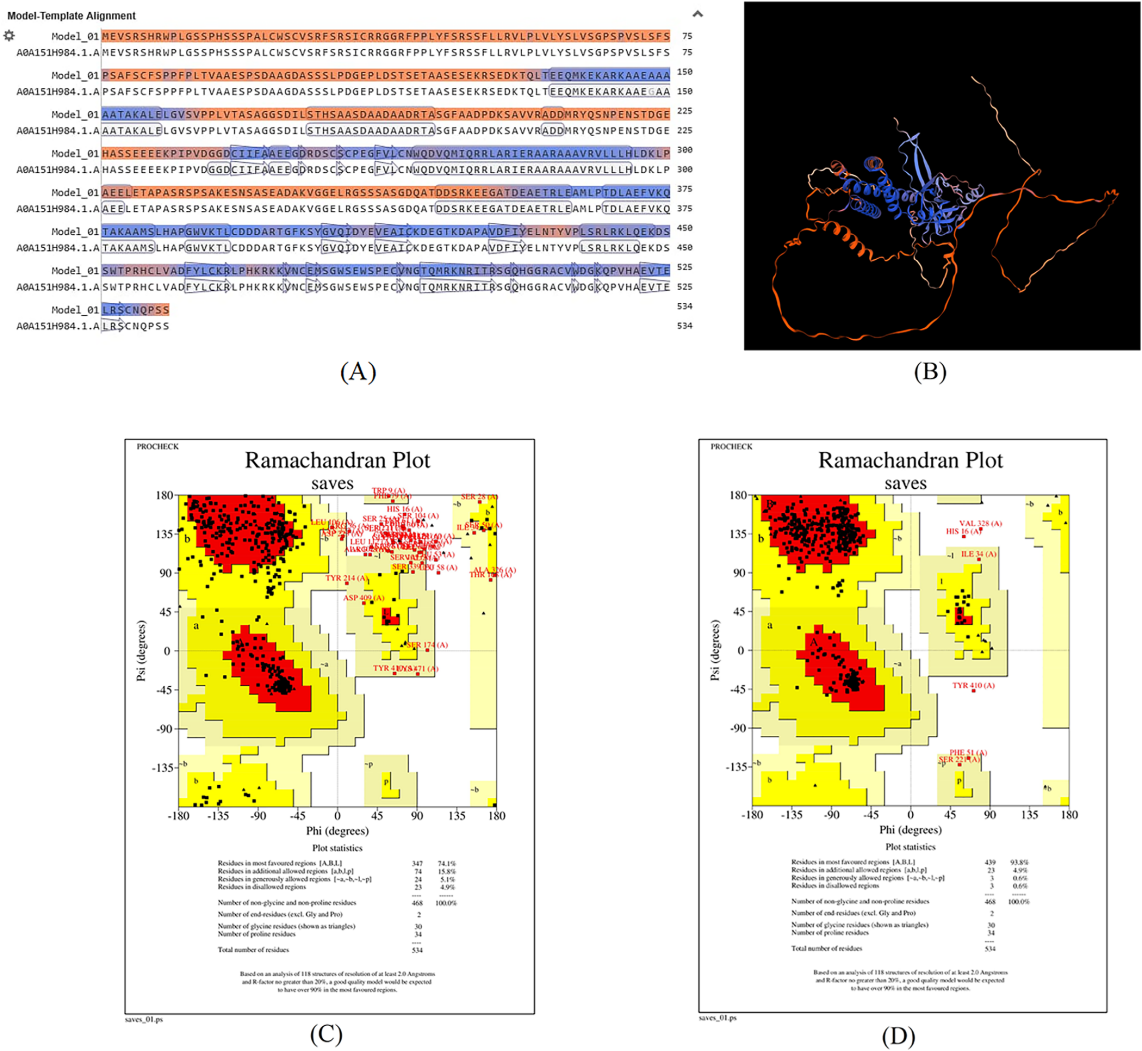

**Figure:**
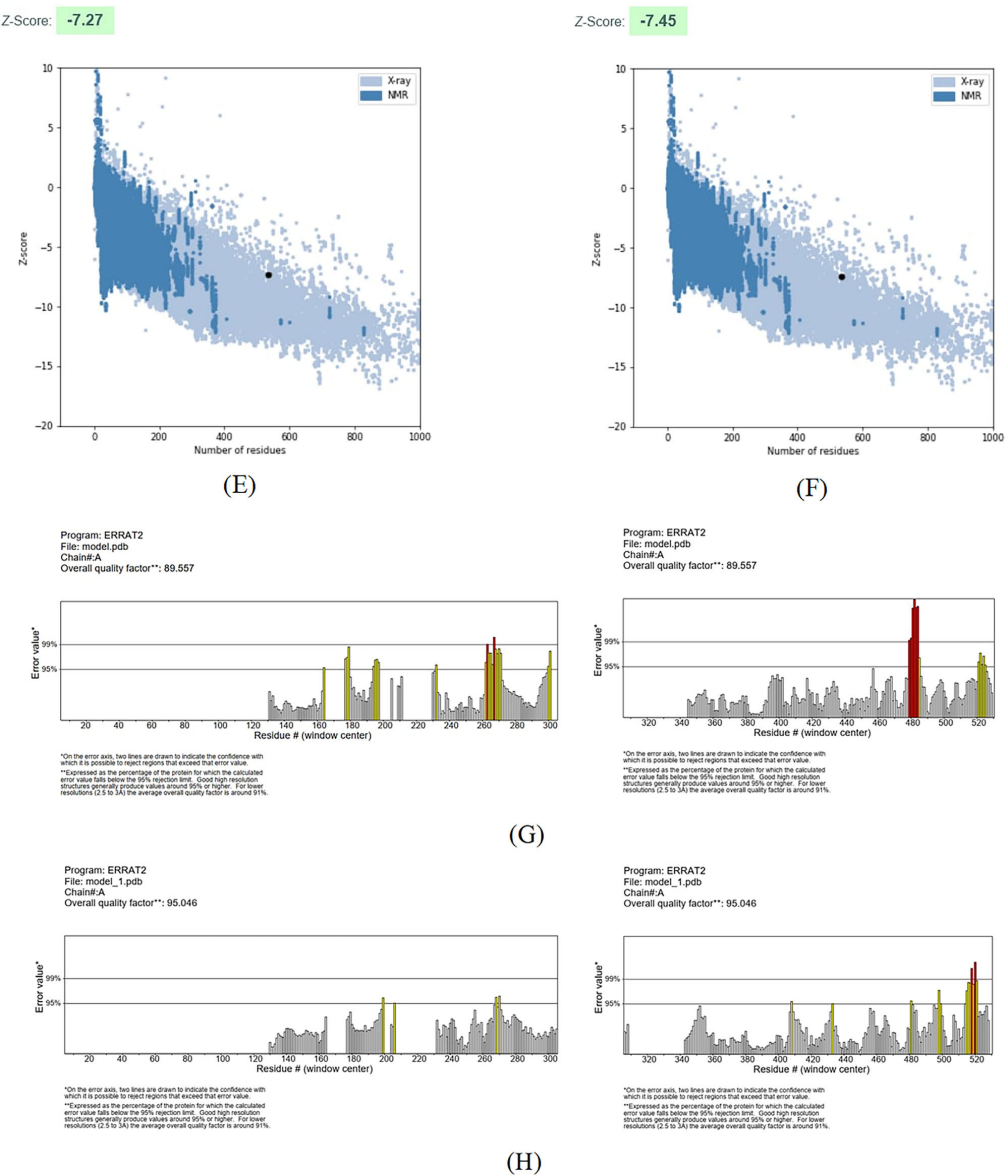

**Figure:**
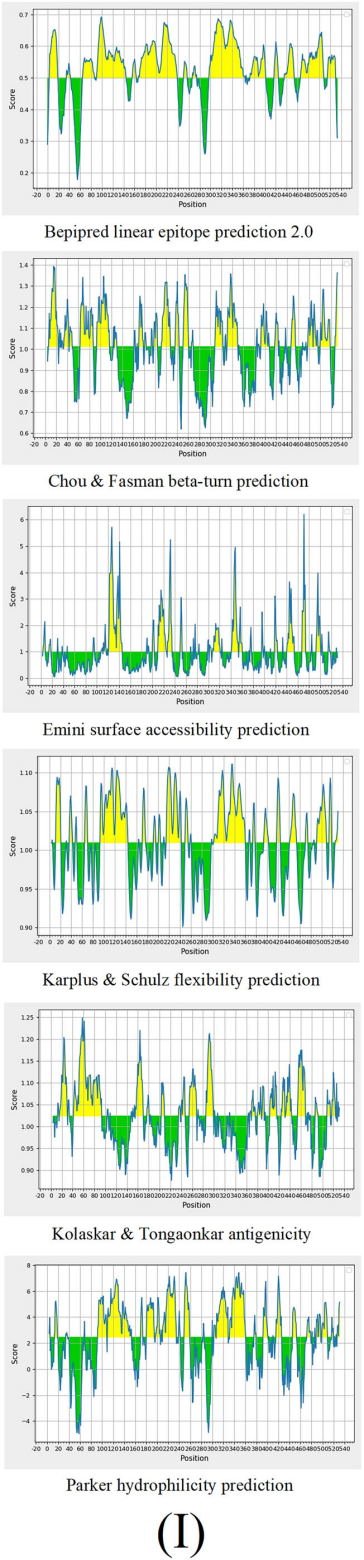

### 3.5 3D Structure Improvement and Verification

The GalaxyRefine tool refined the initial 3D model. The outputs from the GalaxyRefine server for the initial model of TgSPATR were as follows: GDT‐HA = 1.0000, RMSD = 0.0000, MolProbity = 2.353, clash score = 2.1, poor rotamers = 6.2, and Rama favoured percentage = 77.6. After the refinement process, we chose Model one, yielding GDT‐HA, RMSD, MolProbity, clash score, poor rotamers, and Rama favoured values of 0.8553, 0.778, 1.502, 6.4, 0.2, and 97.2, respectively.

SAVES v6.1 validated the 3D models using PROCHECK and ERRAT. A Ramachandran plot for the initial and refined models revealed that 74.1% and 93.8% of the residues were positioned in the favoured regions (Figure 2C,D), indicating an improvement in the 3D model quality of TgSPATR. Furthermore, the ERRAT score improved from 89.557 in the initial model to 95.046 in the refined model (Figure 2G,H), indicating enhanced overall structural quality and fewer potential errors. This improvement supports the predicted stability of the protein's fold, implying that TgSPATR is structurally reliable, making it a suitable candidate for epitope recognition and vaccine design (Colovos and Yeates 1993).

The overall quality of the model was subsequently assessed via the ProSA‐web online tool. The Z scores for the initial and refined models of the TgSPATR protein were estimated to be –7.27 and –7.45, respectively (Figure 2E,F), indicating improved overall 3D structure quality (Wiederstein and Sippl 2007). ProSA‐web (protein structure analysis) is a well‐established application with a large user base that is widely used for modelling and structure prediction as well as for validating and improving experimental protein structures. This tool leverages interactive web‐based applications to present energy plots and scores that highlight potential issues identified in protein structures (Wiederstein and Sippl 2007).

### 3.6 B‐Cell Epitope Prediction

During *Toxoplasma* infection, IgG antibody production is essential to block parasite attachment and facilitate clearance by immune cells (Sayles et al. 2000). The prediction of epitopes offers crucial insights that can aid in the identification of immune‐sensing fragments (Kazi et al. 2018). The secretion of IL‐4 and the activation of a Th‐2 immune response are pivotal in fostering the development and differentiation of B cells (Khan and Moretto 2022; Wang et al. 2016). These mechanisms play a vital role in coordinating a targeted humoral immune response (Sayles et al. 2000).

Linear B‐cell epitopes were predicted using SVMTriP, ABCpred, and BcePred (Tables S1–S3). The specific linear B‐cell epitopes of TgSPATR predicted by the SVMTriP web server are tabulated in Table S1 according to different lengths of 16, 18, and 20 mer. A total of 42 potential linear epitopes were predicted via ABCpred (16 mer = B‐cell epitope length), with scores ranging from 0.75 to 0.95 (Table S2). The BcePred server also aids in predicting B‐cell epitopes on the basis of physicochemical properties in terms of accessibility, exposed surface, turns, polarity, flexibility, antigenic propensity, and hydrophilicity (Table S3). As illustrated in Figure 2I, IEDB analysis provided mean threshold scores for Bepipred linear epitopes, beta‐turns, surface accessibility, flexibility, antigenicity, and hydrophilicity of 0.5, 1.012, 1.000, 1.009, 1.024, and 2.456, respectively. ElliPro predicted 14 conformational B‐cell epitopes with scores of 0.0518–0.917 (Table 2).

**TABLE 2:** The ElliPro was used to predict the conformational B‐cell epitopes of the TgSPATR protein, and a summary of the results is presented below.

No	Residues	No. of residues	Score	3D structure
1	A:E2, A:V3, A:S4, A:R5, A:S6, A:H7	6	0.917	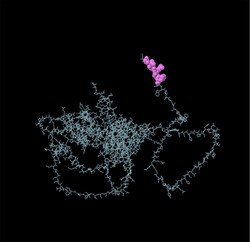
2	A:F46, A:S47, A:R48, A:S49, A:S50, A:F51, A:L52, A:L53, A:R54, A:V55, A:L56, A:P57, A:L58, A:V59, A:L60, A:Y61, A:S62, A:L63, A:V64, A:S65, A:G66, A:P67, A:S68, A:P69, A:V70, A:S71, A:L72, A:S73, A:F74, A:S75, A:P76, A:S77, A:A78, A:F79, A:S80, A:C81, A:F82, A:S83, A:P84, A:P85, A:F86, A:P87, A:L88, A:T89, A:V90, A:A91, A:A92, A:E93, A:S94, A:P95, A:S96, A:D97, A:A98, A:A99, A:G100, A:D101, A:A102, A:S103, A:S104, A:S105, A:L106, A:P107, A:D108, A:G109, A:E110, A:P111, A:L112, A:D113, A:S114, A:T115, A:S116, A:E117, A:T118, A:A119, A:A120, A:S121, A:E122, A:S123, A:E124	79	0.888	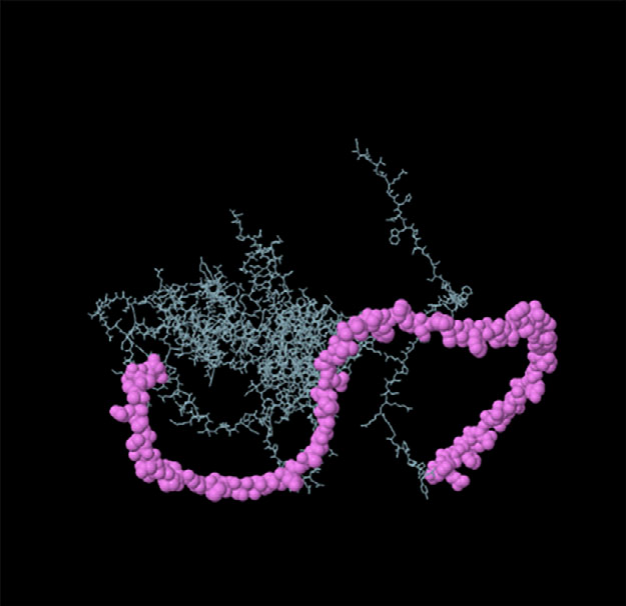
3	A:F41, A:P42, A:P43, A:L44, A:Y45	5	0.771	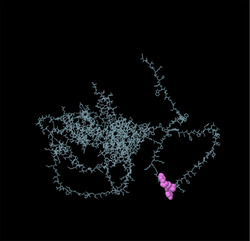
4	A:K125, A:S127, A:E128, A:D129, A:K130, A:T131, A:Q132	7	0.749	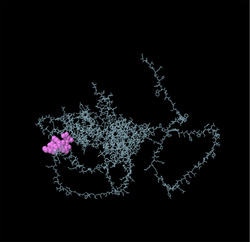
5	A:R8, A:W9, A:P10, A:L11, A:G12, A:S13, A:S14, A:P15, A:H16, A:S17, A:S18, A:S19, A:P20, A:A21, A:L22, A:C23, A:W24, A:S25, A:C26, A:V27, A:S28, A:R29, A:F30, A:S31, A:R32	25	0.749	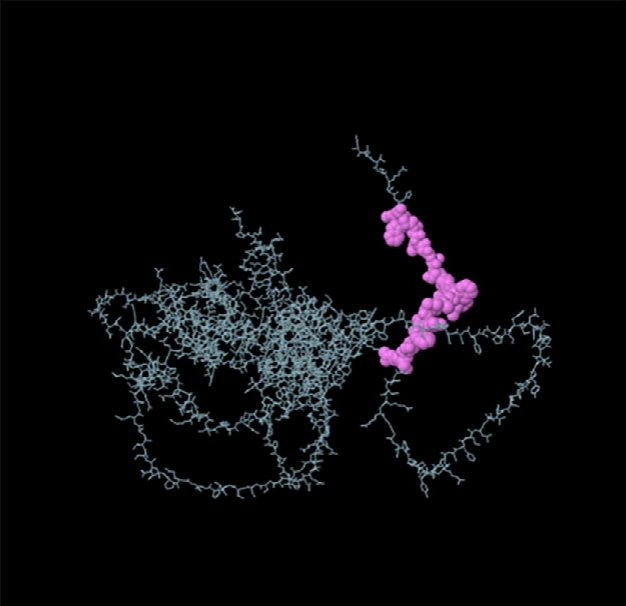
6	A:P308, A:A309, A:S310, A:R311, A:S312, A:P313, A:S314, A:A315, A:K316, A:E317, A:S318, A:N319, A:S320, A:A321, A:S322, A:E323, A:A324, A:D325, A:A326, A:K327, A:V328, A:G329, A:G330, A:E331, A:L332, A:R333, A:G334, A:S335, A:S336, A:S337, A:A338, A:S339	32	0.723	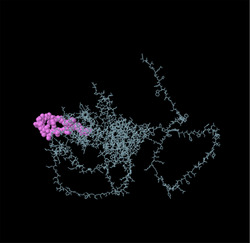
7	A:Y214, A:Q215, A:S216, A:N217, A:P218, A:E219, A:N220, A:S221, A:T222, A:D223, A:G224, A:E225, A:H226, A:A227, A:S228, A:S229, A:E230, A:E231	18	0.717	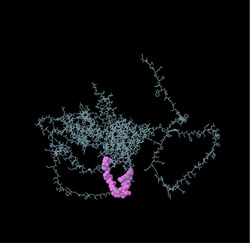
8	A:W482, A:S483, A:E484, A:W485, A:S486, A:P487, A:E488, A:C489, A:V490, A:N491, A:G492, A:T493, A:Q494, A:M495, A:R496, A:E525, A:L526, A:R527, A:S528, A:C529, A:N530, A:Q531, A:P532, A:S533, A:S534	25	0.633	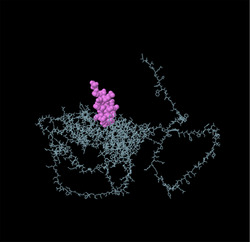
9	A:S33, A:I34, A:C35, A:R36, A:R37, A:G38, A:G39, A:R40	8	0.622	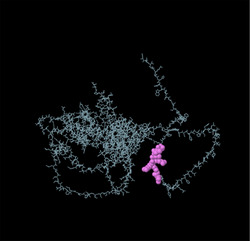
10	A:L133, A:T134, A:E135, A:E136, A:Q137, A:M138	6	0.617	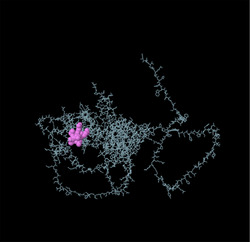
11	A:P165, A:L166, A:V167, A:T168, A:A169, A:S170, A:A171, A:G172, A:G173, A:S174, A:D175, A:I176, A:L177, A:S178, A:T179, A:H180, A:S181, A:A182, A:D185, A:D188, A:A189, A:R192, A:T193, A:L294, A:H295, A:L296, A:D297, A:K298, A:L299, A:P300, A:A301, A:E302, A:E303, A:L304, A:E305, A:T306, A:A307, A:A363, A:M364, A:L365	40	0.583	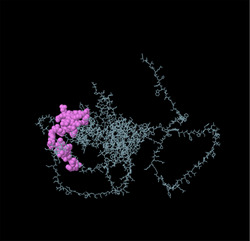
12	A:S195, A:G196, A:F197	3	0.551	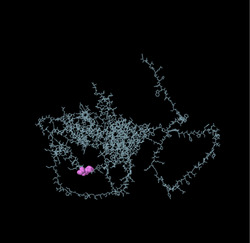
13	A:P438, A:L439, A:S440, A:R443, A:K444, A:E447	6	0.534	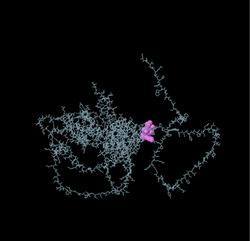
14	A:Q446, A:K448, A:D449, A:S450, A:S451, A:W452, A:V512, A:W513, A:D514, A:G515, A:K516	11	0.518	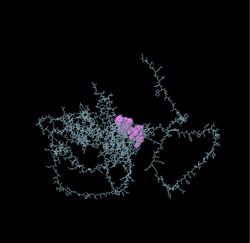

### 3.7 Determination of T‐Cell Epitopes

The binding of peptides to MHC molecules represents a critical step in antigen presentation to T cells and is essential for selecting potential epitopes (Lundegaard et al. 2008b; Wang et al. 2008). IEDB predicted MHC‐I and MHC‐II binding epitopes. The top three epitopes for each allele were selected based on percentile ranks. According to the outputs, TgSPATR can strongly bind MHC molecules, which are crucial for antigen presentation. Tables S4 and S5 list the lowest percentile ranks for MHC‐I and MHC‐II molecules, respectively. The contents of the tables include the alleles used, the potential predicted epitopes, and the percent rank. Various T lymphocytes, including CD4^+^ and CD8^+^ T cells, along with cytokines such as interferon‐γ (IFN‐γ), interleukin‐12 (IL‐12), interleukin‐2 (IL‐2), and tumour necrosis factor‐alpha (TNF‐α), play crucial roles in mediating protective immunity. Additionally, other cytokines, such as IL‐10, IL‐4, and IL‐5, are significant in modulating immune reactions. While CD8^+^ T cells that secrete IFN‐γ are vital for combating toxoplasmosis, the generation of B‐cell antibodies is also critical for long‐term protection against this infection. This immune response enhances the ability to eliminate the *Toxoplasma* parasite and helps prevent reactivation within leukocytes such as macrophages (El‐Kady 2011; Khan and Moretto 2022).

Ten high‐ranking epitopes were selected on the basis of their scores from CTLpred according to the consensus approach. Further details can be found in Table S6.

### 3.8 In Silico Immune Simulation

**FIGURE 3:**
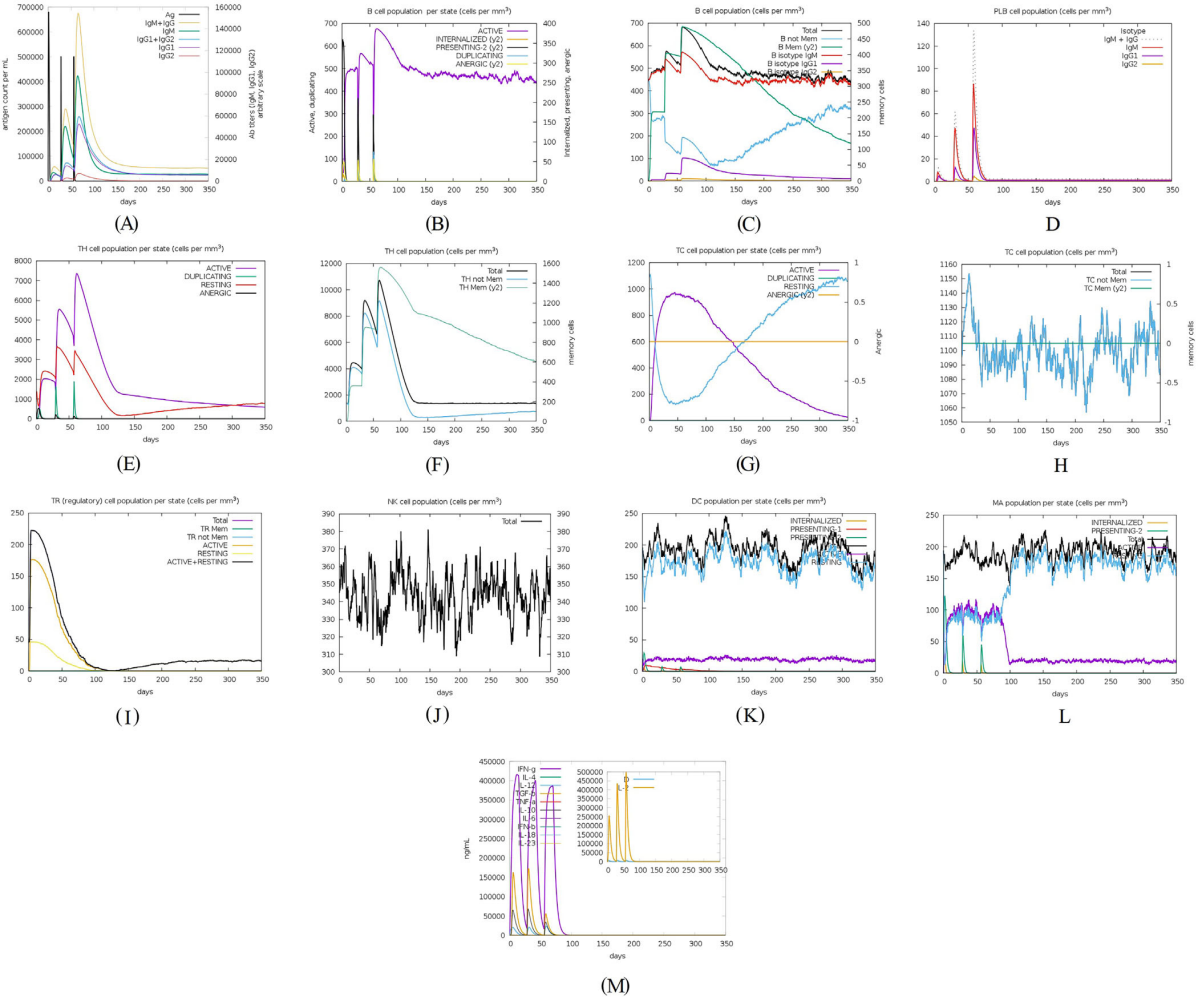
In silico immune simulation in response to *Toxoplasma gondii*‐secreted protein with an altered thrombospondin repeat (TgSPATR). (A) Immunoglobulin production; (B, C) B lymphocyte population; (D) plasma B lymphocytes count sub‐divided per isotype (immunoglobulin M [IgM], immunoglobulin G1 [IgG1], and IgG2); (E, F) T‐helper (TH) cell (CD4^+^) population; (G, H) T‐cytotoxic (TC) cell (CD8^+^) population; (I) T‐regulatory (TR) cell population; (J) natural killer (NK) cell population; (K) dendritic cell (DC) population per state; (L) macrophage population per state; and (M) the level of cytokine production (ng/mL) by TgSPATR.

In silico immune simulation via the C‐ImmSim server is an important step in immunoinformatics‐based studies that helps scientists determine the extent to which a protein can provoke the desired immune response (Rapin et al. 2010). In this study, C‐ImmSim predicted immune responses following three injections at 4‐week intervals. TgSPATR elicited robust humoral responses, especially after the third injection. Specifically, following antigen exposure, substantial levels of IgM (∼100,000) and IgG1 (∼58,000) and a combined measure of both [IgG+IgM] (∼158,000) were observed (Figure 3A). The significant rise in IgM and combined IgG+IgM levels indicates a robust humoral response, which plays a crucial role in controlling infection and establishing protective immunity (Sayles et al. 2000). B‐cell counts peaked at 60 days (∼680 cells/mm^3^) and stabilised at ∼440 cells/mm^3^ over a year, mostly memory B cells (Figure 3B,C). TH‐associated cytokine IFN‐γ (∼400,000 ng/mL) was triggered (Figure 3M). The significant elevation in IFN‐γ, a pivotal cytokine for controlling *T. gondii* infection, further supports the potential of these proteins to elicit protective immune responses (El‐Kady 2011; Khan and Moretto 2022). T‐CD4^+^ and T‐CD8^+^ cells showed prolonged activity (∼1 year for CD4^+^; several weeks for CD8^+^) (Figure 3E–H). NK cell numbers increased for ∼10 days, facilitating IFN‐γ secretion and tachyzoite clearance (Figure 3J). The activation of B cells, TH cells, and TC cells, together with the development of memory B and T lymphocytes, highlights the capacity of the selected protein to induce a broad and durable immune response, which is essential for long‐term protection (El‐Kady 2011; Khan and Moretto 2022; Sayles et al. 2000). Further details are illustrated in Figure 3A–M.

## 4 Limitations

In silico approaches provide an efficient and cost‐effective strategy for the rapid identification of potential antigenic proteins, including those that have not yet been experimentally characterised (Flower et al. 2010; Romano et al. 2011). With ongoing advances in computational algorithms, their role in the preliminary screening of vaccine candidates continues to expand, allowing researchers to prioritise targets more effectively and reduce the experimental burden associated with downstream validation. Despite these advantages, computational predictions must be interpreted with caution. Their reliability is influenced by the completeness of reference databases, underlying algorithmic frameworks, and the lack of biological context (Can et al. 2020; Flower et al. 2010; Kazi et al. 2018). Discrepancies between tools are common—antigenicity, allergenicity, solubility, and immunogenicity scores may vary considerably across prediction servers. For instance, in our previous study, *T. gondii* rhoptry neck protein 4 (TgRON4) was classified as soluble by Protein‐Sol but insoluble by SOLpro, illustrating the type of prediction inconsistency that can occasionally arise in bioinformatics‐based analyses (Foroutan et al. 2025a). Likewise, immune simulation platforms have limited capacity to reproduce the complexity of host responses observed in vivo. Collectively, these limitations underscore that in silico analyses should be regarded as hypothesis‐generating tools rather than definitive evidence. While we emphasise that laboratory validation remains essential, it is important to note that the present work relied solely on in silico analyses. This represents an inherent limitation, as integrating computational predictions with rigorous wet‐lab experiments is ultimately necessary to strengthen vaccine design pipelines and increase the likelihood of identifying truly protective antigens.

## 5 Conclusion

Advances in computer‐based technologies have provided new strategies to prevent infectious diseases. Bioinformatics tools enable the identification of immunoprotective regions, enhancing vaccine development against *T. gondii*. Our study provides insights into TgSPATR, highlighting its potential as a vaccine candidate. The protein exhibited good antigenicity, was non‐allergenic, and contained high‐score B‐ and T‐cell epitopes. Our study demonstrates the value of computational methods in identifying promising antigen candidates, significantly streamlining the early stages of vaccine development. Nevertheless, predictions from in silico analyses require experimental confirmation. Future research should prioritise laboratory validation of TgSPATR, which could be developed as a DNA vaccine, a recombinant protein vaccine, or a multi‐epitope vaccine. Validation should include recombinant protein production, immunogenicity testing, and immunisation experiments in mouse models to assess protective potential. Both acute and chronic phases of infection should be evaluated, including parasite burden in tissues, survival rate, antibody responses, and cytokine profiles. Additionally, optimising antigen delivery platforms and exploring adjuvant combinations could further enhance immune responses. Overall, integrating predictive analyses with empirical studies offers a robust framework for advancing TgSPATR toward a practical and effective vaccine candidate.

## Author Contributions

Conceptualization: MF; Methodology: MF, EKS, and FG; Software: MF and EKS; Validation: MF, EKS, and FG; Formal analysis: MF; Investigation: MF; Resources: MF; Data curation: MF, EKS, and FG; Writing – original draft preparation: MF and EKS; Writing – review and editing: MF and FG; Visualization: MF; Supervision: MF; Project administration: MF; and Funding acquisition: MF. All authors read and approved the final version of the manuscript.

## Funding

Abadan University of Medical Sciences, Abadan, Iran, Grant/Award Number: 1631.

## Disclosure

During the preparation of this work, we used Chat GPT version 5(o) in order to improve the readability and language of the manuscript. After using this, we reviewed and edited the content as needed and took full responsibility for the content of the publication.

## Ethics Statement

This study received approval from the Abadan University of Medical Sciences Ethical Committee (IR.ABADANUMS.REC.1402.009).

## Conflicts of Interest

The authors declare no conflicts of interest.

## Supporting information




**Table S1**: The SVMTriP web server has predicted the specific linear B‐cell epitopes of TgSPATR.

## Data Availability

The data that support the findings of this study are available from the corresponding author upon reasonable request.
