# Supplementary material for: Is Toxoplasma gondii‐secreted Protein With an Altered Thrombospondin Repeat (TgSPATR) a Potential Candidate for Immunisation? An Immunoinformatics‐Based Analysis
Source: Vet Med Sci. 2026 Jan 14;12(1):e70807. doi: 10.1002/vms3.70807 (PMC12800914; doi:10.1002/vms3.70807)
Supplement: Supplementary file 1 — Table S1: The SVMTriP web server has predicted the specific linear B‐cell epitopes of TgSPATR. [file VMS3-12-e70807-s001.docx]

**Table S1.** The SVMTriP web server has predicted the specific linear B-cell epitopes of TgSPATR.

| Rank | Location | Length | Epitope | Score | Recommend* | VaxiJen score ^1^ |
| --- | --- | --- | --- | --- | --- | --- |
| 1 | 359 - 374 | 16 | TRLEAMLPTDLAEFVK | 1.000 | 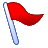 | **1.0526** |
| 2 | 287 - 302 | 16 | AAVRVLLLHLDKLPAE | 0.805 | 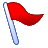 | 0.0252 |
| 3 | 455 - 470 | 16 | RHCLVADFYLCKRLPH | 0.619 |  |  |
| 4 | 432 - 447 | 16 | ELNTYVPLSRLRKLQE | 0.521 |  |  |
| 5 | 21 - 36 | 16 | ALCWSCVSRFSRSICR | 0.466 |  |  |
| 6 | 173 - 188 | 16 | GSDILSTHSAASDAAD | 0.446 |  |  |
| 7 | 42 - 57 | 16 | PPLYFSRSSFLLRVLP | 0.443 |  |  |
| 8 | 155 - 170 | 16 | KALELGVSVPPLVTAS | 0.396 |  |  |
| 9 | 389 - 404 | 16 | VKTLCDDDARTGFKSY | 0.359 |  |  |
| 10 | 104 - 119 | 16 | SSLPDGEPLDSTSETA | 0.350 |  |  |
| 1 | 290 - 307 | 18 | RVLLLHLDKLPAEELETA | 1.000 | 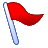 | 0.1553 |
| 2 | 455 - 472 | 18 | RHCLVADFYLCKRLPHKR | 0.928 | 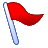 | **1.0023** |
| 3 | 357 - 374 | 18 | AETRLEAMLPTDLAEFVK | 0.849 | 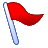 | **1.1178** |
| 4 | 433 - 450 | 18 | LNTYVPLSRLRKLQEKDS | 0.751 |  |  |
| 5 | 389 - 406 | 18 | VKTLCDDDARTGFKSYGV | 0.428 |  |  |
| 6 | 18 - 35 | 18 | SSPALCWSCVSRFSRSIC | 0.414 |  |  |
| 7 | 192 - 209 | 18 | RTASGFAADPDKSAVVRA | 0.411 |  |  |
| 8 | 42 - 59 | 18 | PPLYFSRSSFLLRVLPLV | 0.365 |  |  |
| 9 | 485 - 502 | 18 | WSPECVNGTQMRKNRITR | 0.363 |  |  |
| 10 | 155 - 172 | 18 | KALELGVSVPPLVTASAG | 0.347 |  |  |
| 1 | 132 - 151 | 20 | QLTEEQMKEKARKAAEAAAA | 1.000 | 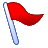 | **1.1113** |
| 2 | 399 - 418 | 20 | TGFKSYGVQIDYEVEAICKD | 0.811 | 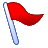 | **0.9462** |
| 3 | 474 - 493 | 20 | KVNCEMSGWSEWSPECVNGT | 0.789 |  |  |
| 4 | 47 - 66 | 20 | SRSSFLLRVLPLVLYSLVSG | 0.689 |  |  |
| 5 | 20 - 39 | 20 | PALCWSCVSRFSRSICRRGG | 0.616 |  |  |
| 6 | 241 - 260 | 20 | GDCIIFAAEEGDRDSCSCPE | 0.599 |  |  |
| 7 | 304 - 323 | 20 | LETAPASRSPSAKESNSASE | 0.592 |  |  |
| 8 | 194 - 213 | 20 | ASGFAADPDKSAVVRADDMR | 0.583 |  |  |
| 9 | 105 - 124 | 20 | SLPDGEPLDSTSETAASESE | 0.513 |  |  |

^1^ VaxiJen score: Threshold for parasites: 0.5

**Table S2.** The ABCpred web service has predicted specific linear B-cell epitopes of TgSPATR, with a threshold of 0.75.

| Rank | Sequence | Start position | Score | VaxiJen score ^1^ |
| --- | --- | --- | --- | --- |
| 1 | SWTPRHCLVADFYLCK | 451 | 0.95 | **0.7204** |
| 1 | SEEEEKPIPVDGGDCI | 229 | 0.95 | 0.3924 |
| 2 | AMSLHAPGWVKTLCDD | 380 | 0.93 | **0.8231** |
| 3 | DRDSCSCPEGFVLCNW | 252 | 0.92 | **1.0699** |
| 4 | GQHGGRACVWDGKQPV | 504 | 0.91 | **0.9343** |
| 4 | PGWVKTLCDDDARTGF | 386 | 0.91 | 0.2947 |
| 5 | ACVWDGKQPVHAEVTE | 510 | 0.90 | **0.7981** |
| 5 | DCIIFAAEEGDRDSCS | 242 | 0.90 | **0.6790** |
| 5 | KSAVVRADDMRYQSNP | 203 | 0.90 | **1.1373** |
| 6 | SGWSEWSPECVNGTQM | 480 | 0.89 | 0.2242 |
| 6 | EVEAICKDEGTKDAPA | 411 | 0.89 | -0.0401 |
| 6 | LETAPASRSPSAKESN | 304 | 0.89 | **0.9002** |
| 6 | VQMIQRRLARIERAAR | 270 | 0.89 | 0.3510 |
| 7 | KNRITRSGQHGGRACV | 497 | 0.88 | **1.7109** |
| 7 | SSASGDQATDDSRKEE | 336 | 0.88 | **1.6251** |
| 8 | LQEKDSSWTPRHCLVA | 445 | 0.86 | 0.4212 |
| 8 | YGVQIDYEVEAICKDE | 404 | 0.86 | **0.8546** |
| 8 | ASEADAKVGGELRGSS | 321 | 0.86 | **1.9998** |
| 8 | STSETAASESEKRSED | 114 | 0.86 | **1.0574** |
| 9 | FYLCKRLPHKRKKVNC | 462 | 0.85 | **0.5625** |
| 10 | ESPSDAAGDASSSLPD | 93 | 0.84 | **1.1249** |
| 10 | SRSICRRGGRFPPLYF | 31 | 0.84 | -0.0303 |
| 11 | RTASGFAADPDKSAVV | 192 | 0.83 | **0.9432** |
| 12 | VSLSFSPSAFSCFSPP | 70 | 0.82 | **0.8509** |
| 12 | SPECVNGTQMRKNRIT | 486 | 0.82 | **0.6925** |
| 13 | GELRGSSSASGDQATD | 330 | 0.81 | **1.7106** |
| 13 | HSSSPALCWSCVSRFS | 16 | 0.81 | -0.6932 |
| 14 | ASESEKRSEDKTQLTE | 120 | 0.80 | **1.3413** |
| 15 | PPLYFSRSSFLLRVLP | 42 | 0.79 | **0.7542** |
| 15 | SAASDAADAADRTASG | 181 | 0.79 | **1.0635** |
| 16 | CFSPPFPLTVAAESPS | 81 | 0.78 | **0.9616** |
| 16 | LSRLRKLQEKDSSWTP | 439 | 0.78 | 0.0547 |
| 16 | AKESNSASEADAKVGG | 315 | 0.78 | **1.5746** |
| 17 | RKAAEAAAAATAKALE | 143 | 0.77 | 0.4124 |
| 18 | YELNTYVPLSRLRKLQ | 431 | 0.76 | 0.4452 |
| 18 | AMLPTDLAEFVKQTAK | 363 | 0.76 | **0.7615** |
| 18 | DDMRYQSNPENSTDGE | 210 | 0.76 | **1.6710** |
| 18 | AAATAKALELGVSVPP | 150 | 0.76 | **0.7449** |
| 19 | AGDASSSLPDGEPLDS | 99 | 0.75 | **1.6094** |
| 19 | PVHAEVTELRSCNQPS | 518 | 0.75 | 0.4438 |
| 19 | EGTKDAPAVDFIYELN | 419 | 0.75 | 0.2086 |
| 19 | QATDDSRKEEGATDEA | 342 | 0.75 | **1.5562** |

^1^ VaxiJen score: Threshold for parasites: 0.5

**Table S3.** The TgSPATR protein epitopes were predicted via many criteria based on the BcePred online application.

| Prediction parameter | Epitope sequence |
| --- | --- |
| Flexibility | MEVSRS, WPLGSSPHSS, SCVSRFSRSICRRGG, DAAGDAS, EPLDSTSETAASESEKRSEDK, EQMKEKA, VTASAGG, DAADRTA, MRYQSNPENSTDGEHASSEEEE, FAAEEGDRDS, TAPASRSPSAKESNSA, KVGGELRGSSSASGDQATDDSRKEE, DDARTGF, AICKDEGT, SRLRKLQEKD, KRLPHKRKK, NGTQMRKNRITRSGQHG, RSCNQPS |
| Hydrophilicity | GSSPHSSSP, AAESPSDAAGDASSS, DGEPLDSTSETAASESEKRSEDKTQLTEEQMKEKARKA, TASAGGSD, THSAASDAADAADRTASG, AADPDKSAV, RYQSNPENSTDGEHASSEEEEKP, AAEEGDRDSCSCPEG, SRSPSAKESNSASEADAKVGGE, RGSSSASGDQATDDSRKEEGATDEAETR, KTLCDDDARTG, CKDEGTKDAPA, KLQEKDSSWT, TRSGQHGGRA, RSCNQPSS |
| Accessibility | EVSRSHRWPL, RRGGRFPP, PDGEPLDSTSETAASESEKRSEDKTQLTEEQMKEKARKAAE, AADPDKSAVVRADDMRYQSNPENSTDGEHASSEEEEKPIPVD, AAEEGDRDS, QMIQRRLARIERAAR, DKLPAEELETAPASRSPSAKESNSASE, GDQATDDSRKEEGATDEAETRLE, VKQTAKAA, DDDARTGFKSY, CKDEGTKDAP, PLSRLRKLQEKDSSWTPRH, CKRLPHKRKKVNCE, NGTQMRKNRITRSGQ, DGKQPVHAE, RSCNQPS |
| Turns | GSSPHSSSPA |
| Exposed Surface | ASESEKRSEDKTQ, TEEQMKEKARKAAE, RYQSNPE, SSEEEEKPIP, TDDSRKEEG, KDEGTKD, SRLRKLQEKDS, CKRLPHKRKKVNCE, TQMRKNRITRS |
| Polarity | MEVSRSHRWPL, SRSICRRGGRFP, AASESEKRSEDKTQLTEEQMKEKARKAAE, VRADDMRY, GEHASSEEEEKPIPV, AAEEGDRDSC, IQRRLARIERAARAA, DKLPAEELETAP, RSPSAKE, ATDDSRKEEGATDEAETRLEAM, KTLCDDDARTG, EAICKDEGTKDA, PLSRLRKLQEKDS, LCKRLPHKRKKVNCEM, TQMRKNRITRS, KQPVHAEVTELRS |
| Antigenic Propensity | LCWSCVSRFSR, FPPLYFS, SSFLLRVLPLVLYSLVSGPSPVSLSFSP, FSCFSPPFPLTV, LELGVSVPPLVT, RDSCSCPEGFVLCNWQ, VRVLLLHLDKLP, GWVKTLCDD, YGVQIDYEV, VDFIYELNTYVPLSRLR, TPRHCLV, FYLCKRLPH, VTELRSC |

**Table S4.** Using the IEDB ^a^, percentile rank values were determined for the affinity of SPATR for MHC class I.

| MHC I allele ^b^ | Start-Stop ^c^ | Peptide sequence | Percentile rank ^d^ |
| --- | --- | --- | --- |
| H2-Db | 61 – 70 | YSLVSGPSPV | 0.16 |
|  | 79 – 88 | FSCFSPPFPL | 0.73 |
|  | 49 – 58 | SSFLLRVLPL | 0.9 |
| H2-Kk | 280 – 289 | IERAARAAAV | 3.2 |
|  | 421 – 430 | TKDAPAVDFI | 3.3 |
|  | 229 – 238 | SEEEEKPIPV | 3.9 |
| H2-Kd | 44 – 53 | LYFSRSSFLL | 1.5 |
|  | 43 – 52 | PLYFSRSSFL | 1.5 |
|  | 430 – 439 | IYELNTYVPL | 2.1 |
| H2-Ld | 453 – 462 | TPRHCLVADF | 1.8 |
|  | 517 – 526 | QPVHAEVTEL | 2.3 |
|  | 18 – 27 | SSPALCWSCV | 5.6 |
| H2-Kb | 77 – 86 | SAFSCFSPPF | 0.2 |
|  | 49 – 58 | SSFLLRVLPL | 0.25 |
|  | 70 – 79 | VSLSFSPSAF | 0.39 |
| H2-Dd | 65 – 74 | SGPSPVSLSF | 0.23 |
|  | 73 – 82 | SFSPSAFSCF | 0.43 |
|  | 37 – 46 | RGGRFPPLYF | 0.88 |

a: The immune epitope database (<http://tools.iedb.org/mhci/>).

b: Mouse MHC class I molecules are H2-Db, H2-Dd, H2-Kb, H2-Kd, H2-Kk, and H2-Ld alleles.

c: Ten amino acids were used for each analysis.

d: High percentile rank = low level binding, low percentile rank = high level binding.

**Table S5.** Using the IEDB ^a^, percentile rank values were determined for the affinity of SPATR for MHC class II.

| MHC II allele ^b^ | Start - Stop ^c^ | Peptide sequence | Percentile rank ^d^ |
| --- | --- | --- | --- |
| H2-IAb | 59 – 73 | VLYSLVSGPSPVSLS | 0.08 |
|  | 60 – 74 | LYSLVSGPSPVSLSF | 0.09 |
|  | 58 – 72 | LVLYSLVSGPSPVSL | 0.09 |
| H2-IAd | 135 – 149 | EEQMKEKARKAAEAA | 0.03 |
|  | 137 – 151 | QMKEKARKAAEAAAA | 0.06 |
|  | 136 – 150 | EQMKEKARKAAEAAA | 0.06 |
| H2-IEd | 461 – 475 | DFYLCKRLPHKRKKV | 0.24 |
|  | 460 – 474 | ADFYLCKRLPHKRKK | 0.25 |
|  | 462 – 476 | FYLCKRLPHKRKKVN | 0.29 |

a: The immune epitope database (<http://tools.iedb.org/mhcii/>).

b: Three mouse MHC class II molecules are known as H2-IAb, H2-IAd, and H2-IEd alleles.

c: Fifteen amino acids were used for each analysis.

d: High percentile rank = low level binding, low percentile rank = high level binding.

**Table S6**. Predicted SPATR epitopes by CTLpred ^a^

| Peptide rank | Start position^b^ | Sequence | Score (ANN/SVM)^c^ | Prediction |
| --- | --- | --- | --- | --- |
| 1 | 288 | AVRVLLLHL | 0.83/0.99156028 | Epitope |
| 2 | 357 | AETRLEAML | 0.71/0.9283511 | Epitope |
| 3 | 267 | WQDVQMIQR | 0.98/0.62775058 | Epitope |
| 4 | 489 | CVNGTQMRK | 0.96/0.60552886 | Epitope |
| 5 | 471 | KRKKVNCEM | 0.71/0.84965854 | Epitope |
| 6 | 276 | RLARIERAA | 0.95/0.5601728 | Epitope |
| 7 | 125 | KRSEDKTQL | 0.96/0.53959595 | Epitope |
| 8 | 450 | SSWTPRHCL | 0.72/0.74380027 | Epitope |
| 9 | 277 | LARIERAAR | 0.92/0.5026669 | Epitope |
| 10 | 50 | SFLLRVLPL | 0.98/0.39142527 | Epitope |

^a^ CTLpred, available online at <http://crdd.osdd.net/raghava/ctlpred/>.

^b^ Nine amino acids were used for analysis.

^c^ The default artificial neural network (ANN) and support vector machine (SVM) cutoff scores were set to 0.51 and 0.36, respectively.
